# Book Review: Language Teacher Cognition: A Sociocultural Perspective

**DOI:** 10.3389/fpsyg.2020.01098

**Published:** 2020-06-12

**Authors:** Qiaoying Cai, Xinmin Zheng

**Affiliations:** ^1^Institute of Linguistics, Shanghai International Studies University, Shanghai, China; ^2^College of Foreign Languages, Fujian Normal University, Fujian, China; ^3^School of Education, Shanghai International Studies University, Shanghai, China

**Keywords:** language teacher cognition, sociocultural, teacher education, interactive decision making, identity, emotion, the use of technology in education

Written by Li Li, *Language Teacher Cognition: A Sociocultural Perspective*, as the title indicates, explores the topic of teacher cognition from the perspective of sociocultural theory, aiming to theorize and analyze what teachers think, believe, know, and do in their professional contexts through the lens of social interaction and Vygotsky's theory of mental development. This book was published in October 2019 as an extension and expansion of the author's previous book (Li, 2017), answering (Johnson, 2009) call for a social turn for teacher cognition stressing the importance of teacher learning in communities, and how their experiences can help them develop professional thinking and understanding (Li, 2020).

This book consists of nine chapters. The first *chapter* provides readers with a brief review of language teacher cognition research, lays out the theoretical underpinning for this book, i.e., the combination of sociocultural theory (SCT), discursive psychology (DP), and conversation analysis (CA), and illustrates the significance of this book and its intended readers.

The *second chapter* discusses the significance of language teacher cognition research and its many different theoretical understandings and research methods, offering a profound and new understanding of the key concepts of SCT, i.e., mediation, ZPD (zone of proximal development), and internalization.

The *third chapter* elaborates on the link between teacher cognition and social interaction, demonstrating how interaction with others embodies teachers' understanding, knowledge, conceptualization, and beliefs, providing readers with an understanding of the rationale of CA including its principles, turn taking, adjacency pairs and preferences, repair, and how to handle data in terms of recording and transcription.

*Chapter 4* presents the conceptions and knowledge teachers have on teaching and learning, drawing from data collected from Chinese English classrooms.

*Chapter 5* investigates the macro interactive decisions that teachers make on a moment-by-moment basis in four different situations: unexpected or dis-preferred responses from learners, unexpected incidents arising in the tasks and activities, insufficient subject knowledge of teachers, and learning opportunities emerging in the situations.

*Chapter 6* focuses on teacher cognition regarding teachers' use of technology to meet their pedagogical needs, analyzing teachers' accounts of using technology and their practice of technology through the lens of CA.

*Chapter 7* evaluates the impact of teacher education on teacher cognition, illustrating that pre-service teachers' beliefs are not stable and could be changed, which is in contrary to the existing research findings that student teachers' beliefs are deeply rooted and not easy to change.

*Chapter 8* is devoted to a hotword in teacher research in the twenty-first century—teacher identity, bringing in the key elements in relation to the concept of identity: the “investment” theory, imagined communities, agency, and conception self, emotion, and the important factors influencing professional identity formation.

The last chapter, *chapter 9*, serves as a closing chapter, which is built on the previous ones to generate key themes regarding teacher cognition, the implications of teacher cognition in teacher education and development.

Just as the author writes in the closing remarks, by exemplifying and scrutinizing the critical ideas in sociocultural theory and their implications for teacher cognition, this book has added significant value to the existing research on teacher education and development (Li, 2020), in that it paints a grand, yet fine, picture of teacher cognition covering the most important as well as the most updated themes regarding teacher cognition through the lens of sociocultural theory, under a well-established theoretical framework supported with abundant first-hand data collected in the front-line language classrooms. With a brief review of language teacher research at the outset, the author lays the backdrop for the whole book, and with the combination of detailed explanation of key concepts at the beginning of each chapter and exemplified illustrations with data collected in real-time classrooms, the author offers readers a holistic view of each key theme discussed in the chapters and engages readers up to the end. Closing the book, readers are left with a well-marked map for the wonderful world of language teacher cognition.

As Kubanyiova and Feryok (2015) suggested, despite the rapidly expanding research activity in the field of language teacher cognition, the current studies have not been able to answer some of the most essential questions: how do language teachers create meaningful learning environments for their students (Kubanyiova and Feryok, 2015)? How can teacher education facilitate such learning (Kubanyiova and Feryok, 2015)? Only by viewing teacher cognition through an alternative lens and embracing the sociocultural turn in this field can relationships between teachers' cognition, practice and students' learning be rightly examined (Kubanyiova and Feryok, 2015). This book is a timely and thorough report on the frontier research in the field, and its biggest significance lies in the sociocultural perspective it takes when examining teacher cognition, discarding the conventional top–down approach in exchange for a bottom–up approach by taking the situation and contexts where teachers interact with themselves and others into account.

It would be more comprehensive if the book could include the study of teacher cognition and practice about online teaching and learning, or blended teaching methods since online teaching and learning is on the rise and is occupying an increasingly large proportion of the educational sector whose impact on teacher cognition and practice could not be ignored.

This book should have a broad audience including researchers who are already working in this field, anyone who are interested in interaction, doctoral and master's students, as well as teachers. As researchers in the field of language teacher cognition ourselves, we find this book attractive, well-organized, fully illustrated, easy to read, and exceedingly informing and would highly recommend it to anyone who is interested in language teacher cognition.

## Author Contributions

QC and XZ chose the book together. QC wrote the review. XZ provided valuable ideas for the drafts.

## Conflict of Interest

The authors declare that the research was conducted in the absence of any commercial or financial relationships that could be construed as a potential conflict of interest.
